# Acceptance of Internet-Based Health Care Services Among Households in Poland: Secondary Analysis of a Population-Based Survey

**DOI:** 10.2196/jmir.2358

**Published:** 2012-11-27

**Authors:** Mariusz Duplaga

**Affiliations:** ^1^Institute of Public HealthJagiellonian University Medical CollegeKrakowPoland

**Keywords:** eHealth, health care service, Internet use, computer use

## Abstract

**Background:**

Polish society is benefiting from growing access to the Internet, but the use of advanced e-services is still limited. The provision of Internet-based health services depends not only on the penetration of the Internet into society, but also on the acceptance of this technology by potential users.

**Objective:**

The main objective of this study was focused on the assessment of predictors of acceptance of Internet use for provision of health services (eg, sociodemographic status, the use of information technologies, and consumption of health care services) among households in Poland.

**Methods:**

The study was based on a secondary analysis of the dataset from the 2011 Social Diagnosis survey (a biannual survey conducted since 2001 about economic and non-economic aspects of household and individual living conditions in Poland). Analysis of the questionnaire results focused on the situations of the households included in the study. The predictors for 2 outcome variables describing the acceptance of households for Internet use for provision of a full health care service, or at least access to information and download of required forms, were assessed using multivariate logistic regression.

**Results:**

After excluding those households that would not consider the use of health care services or for which predictor variables assumed missing values, the final analyses were conducted on data from 8915 households. Acceptance of the use of the Internet for provision of full health care services in Polish households was significantly higher among households in urban locations with ≥ 200,000 inhabitants than among households in rural areas; it was also higher with salaried employment as the source of income than with self-employment in agriculture (odds ratio [OR] = 0.53, 95% CI 0.40 - 0.70), retirement pension (OR = 0.46, 95% CI 0.39 - 0.54), disability pension (OR = 0.48, 95% CI 0.34 - 0.68), or with several simultaneous income sources (OR = 0.66; 95% CI 0.57 - 0.79). Furthermore, acceptance of Internet-based health care was higher in households with a higher monthly net income per capita (OR = 2.11, 95% CI 1.75 - 2.53 for households from the lowest and the highest income interval), among households with > 1 child aged < 15 years (OR = 1.38, 95% CI 1.20 - 1.59), among households with at least some books (with OR = 3.33, 95% CI 2.39 - 4.64 for household with no books and those with over 500 books). Acceptance was also higher in households with a computer (OR = 1.86, 95% CI 1.35 - 2.56), Internet access (OR = 1.95, 95% CI 1.37 - 2.76), and Internet access for a longer duration (OR = 1.36, 95% CI 1.06 - 1.75 and OR = 1.81, 95% CI 1.40 - 2.33 for households with access < 1 year versus those with access for 1-5 years and > 5 years, respectively). Greater self-declared confidence in using technology was also associated with higher acceptance of the Internet for health care services (OR = 2.94, 95% CI 2.21 - 3.91 for the least confident households versus those with the highest confidence). Furthermore, recent use of health care services increased acceptance of using the Internet for at least some health-related services (OR = 1.49, 95% CI 1.16 - 1.91), but not for full provision of online health care services (OR = 1.20, 95% CI 0.92 - 1.55). Neither the hospitalization of a member of a household nor the opinion about satisfying health care needs of a household affected the degree of acceptance.

**Conclusions:**

The acceptance of health care services through the Internet is higher in households from larger cities, with stable income from an employee salary, as well as with higher income levels per capita. Furthermore, general computer and Internet use in the household influenced the perception of eHealth. Paradoxically, the use of health care services or the level of satisfaction with the coverage of the household’s health needs has a limited influence on acceptance of Internet-based health care services.

## Introduction

Internet use has increased considerably in Poland over the past decade. The percentage of households with Internet access when Poland joined the European Union (EU) in 2004 was 26% [1]. According to the statistical office of the European Community (Eurostat), it reached 62% in 2011 [2]. The accession to the EU opened new prospects for the development of e-services, mainly due to the structural funds and special programs available for supporting business models and administrative services based on electronic communication [3-6]. Although there is a visible growth trend in the information society in Poland, the availability of advanced e-services differs considerably between specific domains. This applies particularly to the field of eHealth, which shows relatively slow progress [7]. Apart from access to information about health and health care, the more advanced eHealth services, such as telemonitoring or Internet-based appointment bookings with a physician, are not commonly used [8,9]. Some eHealth services are not available due to legal restrictions (eg, e-prescriptions) although they are becoming increasingly common in other EU member countries. Furthermore, there are no established reimbursement schemes for eHealth services in the publicly funded health care system in Poland.

User acceptance is a key condition for wide implementation of innovative information and communication technology (ICT) solutions, including eHealth [10]. So far, awareness and acceptance of eHealth among Polish citizens has not been studied thoroughly. According to a survey by Staniszewski et al [11] in 2007, the percentage of respondents who declared that they are familiar with the term *telemedicine* was only 32.1%. The awareness of the term *eHealth* was not surveyed. This is understandable because eHealth is rarely used in the media, although it is used (instead of *telemedicine* or *health ICT*) in policy documents issued by governmental bodies [12]. The main focus of most surveys to date has been the use of the Internet for the conduct of health-related activities [2,11]. The opinion of Polish households about the use of the Internet for specific activities has been surveyed in recent versions of the Social Diagnosis study (a biannual survey conducted since 2001 about economic and non-economic aspects of household and individual living conditions in Poland). The number of households that expressed acceptance of the full delivery of health care services online has not significantly changed since 2007. In 2007, acceptance was approximately 30% and it has remained stable since then [13].

The main goal of this study was a secondary analysis of data collected during the most recent Social Diagnosis study (2011). The scope of the study is broad and covers many areas, including household living conditions, individual quality of life, the state of civil society and economic status, the usage of new communication technology, and social exclusion. The methodology and primary analysis of the collected data was published by Czapinski et al [14]. The database containing the survey results is publicly available [15]. The focus of our study was the assessment of possible predictors for the household acceptance of the use of the Internet for provision of health care services in Poland.

## Methods

The data included in the current analysis were obtained from households included in the Social Diagnosis survey in 2011. The data were collected using 2 questionnaires. The first questionnaire, covering household structure and living conditions, and the sociodemographic characteristics of its members, was completed by professional canvassers employed by the Central Statistical Office who interviewed the household representatives with the most complete knowledge of their circumstances. The second questionnaire was designed to be completed by all household members aged ≥ 16 years. The selection of households for participation in the survey was the result of a 2-level stratified sampling. It was preceded by stratification of households according to *voivodeships* (main territorial unit in Poland roughly corresponding to state or county in other countries), and then within voivodeships according to area of residence (eg, rural or urban). A detailed description of the sampling procedure is given in the relevant report on the study [16]. The structure of the questionnaires used for the survey may be viewed on the study website [17].

The analysis described in this paper was conducted on data originating in the questionnaire about household circumstances. In section M of the questionnaire, the items related to the use of the Internet to accomplish specific types of services were included, such as vehicle registration, handling cases related to personal documents (eg, identity cards), and business activities. One of the items enquired about the household’s view on using the Internet to provide health-related or health care services.

The household representative answering this item could select one of 4 responses: (1) “I do not need the Internet for this service,” (2) “I would like to obtain information or download the required forms online and then proceed in the traditional way,” (3) “I would like to be able to complete the entire transaction online (including payment),” and (4) “I do not anticipate the use of such a service.” Only cases with a valid response to this item were extracted from the database containing all the data collected in 2011 and used for further analyses (12,015 of 12,386 households) [15].

Two outcome variables for this item were defined for the logistic regression analysis. The first outcome variable assumed the value *yes* if the respondent selected option 3 and the value *no* if they selected option 1 or 2. The second outcome variable assumed the value *yes* if option 2 or 3 was chosen and the value *no* for option 1. Cases with option 4 chosen as a response were excluded from the analysis. A total of 10,315 cases were included for further analysis.

From the 2011 data, 14 variables were derived and included in the logistic regression procedure as predictors. The variables were selected according to their potential influence on the acceptance of the use of the Internet for health care services.

Statistical analysis was conducted using the Epi Info version 3.5.4 software (Epi Info, Centers for Disease Control and Prevention, Atlanta, GA, USA). Descriptive statistics were calculated for all variables. Logistic regression was performed in order to assess potential predictors for the acceptance of the use of the Internet for provision of health care services. All cases with missing values (1400/10,315) for any of the predictor variables included in the model were excluded from the analysis. Finally, multivariate logistic regression was calculated on the dataset of 8915 cases.

## Results

### Characteristics of the Sample of Households

The frequencies for the categorical variables are shown in Table 1. The sample included 39.81% households from rural areas. Furthermore, income from employment was the main source of income for 44.60% of households, retirement pension for 28.03%, and more than 1 type of income for 10.32% of households. There was 1 child aged < 15 years in 17.51%, 2 in 10.85%, and > 2 in 3.50% of households. The economic status as assessed by a household representative improved in comparison to the status 2 years previously in only 10.79% of households, and remained unchanged in 56.87%. A total of 10.84% of households received some form of external support. The number of books in the household was > 100 in only 22.25% of households.

**Table 1:** The characteristics of households included in the multivariate logistic regression analysis (N = 8915).

Characteristic		n	%
**Acceptance of the Internet use for provision of health care services (variant I: provision of full service)**			
	No	6259	70.21
	Yes	2656	29.79
**Acceptance of the Internet for provision of health service (variant II: access to information/forms or full service provision)**			
	No	4895	54.91
	Yes	4020	45.09
**Place of residence (number of inhabitants)**			
	Rural	3549	39.81
	Urban <20,000	1167	13.09
	Urban 20,000-100,000	1803	20.22
	Urban 100,000-200,000	611	6.85
	Urban 200,000-500,000	926	10.39
	Urban >500,000	859	9.64
**Source of household income**			
	Employment (wages and salaries)	3976	44.60
	Self-employment in agriculture	404	4.53
	Self-employment outside agriculture	384	4.31
	Retirement pension	2499	28.03
	Disability pension	468	5.25
	Non-employment source other than retirement or disability pensions	264	2.96
	Numerous parallel income sources	920	10.32
**Monthly household net income per capita, Polish zloty (PLN)** ^a^			
	< 700	2106	23.62
	≥ 700 and < 1000	2284	25.62
	≥ 1000 and < 1500	2101	23.57
	≥ 1500	2424	27.19
**Opinion about economic status of household compared to 2 years ago**			
	Worsened	2883	32.34
	Unchanged	5070	56.87
	Improved	962	10.79
**Reception of aid from external sources**			
	No	7949	89.16
	Yes	966	10.84
**Number of children aged < 15 years in household**			
	0	6075	68.14
	1	1561	17.51
	2	967	10.85
	>2	312	3.50
**Number of books in household**			
	None	1007	11.30
	≤ 25	2034	22.81
	26-50	2028	22.75
	51-100	1862	20.89
	101-500	1505	16.88
	>500	479	5.37
**Computer in household (PC or notebook)** ^b^			
	No	3041	34.11
	Yes	5874	65.88
**Internet access in household**			
	No	3448	38.68
	Yes	5467	61.32
**Duration of Internet access**			
	< 1 year	3693	41.42
	1-5 years	2940	32.98
	>5 years	2282	25.60
**Opinion about being up-to-date with modern technology**			
	Strongly disagree	2450	27.48
	Somewhat disagree	2496	28.00
	Neither agree nor disagree	1710	19.18
	Somewhat agree	1922	21.56
	Strongly agree	337	3.78
**Use of health care services in the last year** ^c^			
	No	419	4.70
	Yes	8496	95.30
**Hospital admission of a household member household in the preceding 12 months** ^d^			
	No	6519	73.12
	Yes	2396	26.88
**Opinion about satisfying of health needs of a household in comparison to the situation 2 years ago**			
	Worsened	2391	26.82
	Unchanged	6309	70.77
	Improved	215	2.41

^a^ The median and quartile values of monthly household net income per capita were calculated for the initial set of 12,015 households with valid data on the acceptance of Internet use for the provision of health services (median 1000 Polish zlotys [PLN], lower quartile 700 PLN, and upper quartile 1500 PLN). These values were used to determine 4 intervals for categorizing monthly household net income per capita. 1 PLN = US $0.31 (mid-market rate November 19, 2012).

^b^ Yes: at least one member of the household owned a personal computer or mobile computer (eg, notebook, laptop, iPad, or tablet); no: no personal or mobile computer in a household.

^c^ Yes: the household used health care services funded by the National Health Fund or paid out of pocket or paid by employer in past year; no: the household did not use health care services in the past year.

^d^ Yes: at least one member of the household was admitted to hospital in past year; no: no hospitalization of members of household in past year.

Regarding computer use, 65.88% of households had a personal or mobile computer, and 61.32% had Internet access. Only 25.60% of households had Internet access for more than 5 years. Approximately one-quarter of households (25.34%, 2259/8915) felt that they were “up-to-date with modern technologies.”

Most households (95.30%) declared that their members used health care services with 26.88% having members of their household admitted to hospital in the preceding year. In the opinion of 70.77% of households, the coverage of their health needs had not changed in comparison with 2 years ago, and had improved in only 2.41% of households.

Less than half of the households (45.09%) included in the analysis accepted Internet use for full or at least partial (access to information and document download) delivery of health care services. The percentage of households expressing an opinion in favor of Internet use for complete provision of health care services was 29.79% (2656/8915).

### Factors Related to the Acceptance of the Use of the Internet for Health Care Services

The results of the analysis revealed that predictors of the acceptance of the use of the Internet for full provision of health care services included: monthly household income per capita, place of residence, number of children aged < 15 years, source of income, reception of aid from external sources (social care), availability of a computer (PC or mobile) in a household, Internet access and its duration, opinion about being up-to-date with modern technologies, and the number of books in the household.

The acceptance of the use of the Internet for full health care services, or for at least access to information and downloading documents, was predicted by the same variables and additionally by the use of health care services during the past year. The odds ratios (ORs), confidence intervals (CIs), and *P* values resulting from the multivariate logistic regression are presented in Table 2.

**Table 2:** The results of multivariate logistic regression model for factors affecting the acceptance of Internet-based health care services.

Variable		Acceptance of reception of health services by the Internet			
		Full service provision		Full service provision or only access to information and forms	
		Odds ratio (95% CI)	*P*	Odds ratio (95% CI)	*P*
**Place of residence**			< .001		< .001
	Rural	1		1	
	Urban < 20,000	0.92 (0.77 - 1.09)	.33	0.93 (0.79 - 1.09)	.36
	Urban 20,000-100,000	1.13 (0.97 - 1.31)	.11	1.23 (1.06 - 1.42)	.005
	Urban 100,000-200,000	0.96 (0.77 - 1.19)	.68	1.13 (0.92 - 1.40)	.24
	Urban 200,000-500,000	1.57 (1.31 - 1.89)	< .001	1.63 (1.35 - 1.97)	< .001
	Urban > 500,000	1.96 (1.61 - 2.37)	< .001	2.42 (1.97 - 2.97)	< .001
**Source of household income**			< .001		< .001
	Employment (wages and salaries)	1		1	
	Self-employment in agriculture	0.53 (0.40 - 0.70)	< .001	0.61 (0.48 - 0.78)	< .001
	Self - employment outside agriculture	0.94 (0.75 - 1.19)	.62	1.04 (0.81 - 1.34)	.74
	Retirement pension	0.46 (0.39 - 0.54)	< .001	0.47 (0.40 - 0.54)	< .001
	Disability pension	0.48 (0.34 - 0.68)	< .001	0.53 (0.40 - 0.71)	< .001
	Non-employment source other than retirement pensions or disability payment	1.05 (0.74 - 1.48)	.79	0.99 (0.72 - 1.37)	.97
	Numerous parallel income sources	0.66 (0.57 - 0.79)	< .001	0.70 (0.60 - 0.83)	< .001
**Monthly household net income per capita (PLN)**			< .001		< .001
	< 700	1		1	
	≥ 700 and < 1000	1.38 (1.17 - 1.63)	< .001	1.25 (1.08 - 1.49)	.004
	≥ 1000 and < 1500	1.62 (1.36 - 1.94)	< .001	1.41 (1.19 - 1.67)	< .001
	≥ 1500	2.11 (1.75 - 2.53)	< .001	1.70 (1.42 - 2.03)	< .001
**Economic status of household compared to 2 years ago**			.56		.93
	Worsened	1		1	
	Unchanged	0.84 (0.74 - 0.95)	.009	0.78 (0.69 - 0.88)	< .001
	Improved	0.98 (0.81 - 1.17)	.79	1.12 (0.93 - 1.36)	.23
**Reception of aid from external sources**			.02		.045
	No	1		1	
	Yes	1.18 (0.97 - 1.44)	.09	1.14 (0.95 - 1.37)	.16
**Number of children aged < 15 years**			< .001		< .001
	0	1		1	
	1	1.38 (1.20 - 1.59)	< .001	1.38 (1.20 - 1.59)	< .001
	2	1.33 (1.12 - 1.58)	.001	1.38 (1.16 - 1.64)	< .001
	> 2	1.41 (1.06 - 1.88)	.018	1.31 (1.00 - 1.73)	.05
**Number of books in household**			< .001		< .001
	None	1		1	
	≤ 25	1.46 (1.11 - 1.92)	.007	1.38 (1.10 - 1.74)	.005
	26-50	1.47 (1.12 - 1.93)	.006	1.59 (1.26 - 1.99)	< .001
	51-100	1.72 (1.30 - 2.26)	< .001	1.91 (1.52 - 2.40)	< .001
	101-500	2.07 (1.56 - 2.74)	< .001	2.36 (1.86 - 3.01)	< .001
	> 500	3.33 (2.39 - 4.64)	< .001	3.46 (2.52 - 4.75)	< .001
**Computer in household**			< .001		< .001
	No	1		1	
	Yes	1.86 (1.35 - 2.56)	< .001	2.14 (1.66 - 2.76)	< .001
**Internet access in household**			< .001		< .001
	No	1		1	
	Yes	1.95 (1.37 - 2.76)	< .001	1.72 (1.27 - 2.32)	< .001
**Duration of Internet access**			< .001		< .001
	< 1 year	1		1	
	1-5 years	1.36 (1.06 - 1.75)	.02	1.59 (1.26 - 2.01)	< .001
	> 5 years	1.81 (1.40 - 2.33)	< .001	2.02 (1.58 - 2.57)	< .001
**Consider self up-to-date with modern technology**			< .001		< .001
	Strongly disagree	1		1	
	Rather disagree	1.31 (1.11 - 1.55)	.002	1.23 (1.06 - 1.43)	.006
	Neither agree nor disagree	1.67 (1.40 - 2.00)	< .001	1.78 (1.52 - 2.09)	< .001
	Rather agree	1.77 (1.49 - 2.10)	< .001	1.73 (1.47 - 2.04)	< .001
	Strongly agree	2.94 (2.21 - 3.91)	< .001	2.92 (2.13 - 4.00)	< .001
**Use of health care services in last year**			.22		.002
	No	1		1	
	Yes	1.20 (0.92 - 1.55)	.18	1.49 (1.16 - 1.91)	.002
**Hospital admission in last year**			.60		.31
	No	1		1	
	Yes	1.05 (0.93 - 1.18)	.47	0.95 (0.85 - 1.07)	.41
**Opinion about satisfaction of health needs of a household**			.59		.95
	Worsened	1		1	
	Unchanged	1.00 (0.88 - 1.15)	.95	1.01 (0.89 - 1.14)	.92
	Improved	1.22 (0.87 - 1.70)	.25	1.07 (0.75 - 1.52)	.73

Households from urban areas with at least 200,000 inhabitants were more likely to accept the use of the Internet for health care services (for both variants of the outcome variable). In addition, households with a retirement or illness pension, farmer’s income, or several sources of income were less inclined to accept the use of the Internet for this purpose than those with an employee’s salary as the main source of income. Internet acceptance also depended on monthly household net income per capita, with growing acceptance at higher income levels (in comparison to values below the lower quartile). The OR for the outcome variable assuming acceptance of full health care services provided online were 1.38 (95% CI 1.17 - 1.63), 1.62 (95% CI 1.36 - 1.94), and 2.11 (95% CI 1.75 - 2.53), respectively for income levels.

The presence of 1 or 2 children aged < 15 years increased Internet acceptance of health care services in comparison to households without children in that age range. The values of OR for full provision of the service in the Internet were 1.38 (95% CI 1.20 - 1.59), 1.33 (95% CI 1.12 - 1.58), and 1.41 (95% CI 1.06 - 1.88) for 1, 2, and > 2 children in a household, respectively.

The reception of aid from external services, presumably from social care, was associated with both outcome variables in the general multivariate regression model. However, in the model with specified dummy variables derived from the main variables, this significant relationship was not maintained.

Households with at least some books revealed a higher acceptance of the use of the Internet for health care provision in comparison to households with no books at all. This relationship was valid for both outcome variables. Both outcome variables showed a significant association with the availability of a personal or mobile computer in a household, with OR = 1.86 (95% CI 1.35 - 2.56) and OR = 2.14 (95% CI 1.66 - 2.76) for full and at least partial acceptance of the Internet for the provision of health care services, respectively. Access to the Internet and duration of Internet access lasting at least 1 year increased the probability of acceptance of full online health care services in comparison to households without Internet access or access of less than 1 year.

Self-confidence in being up-to-date with modern technologies was associated with higher acceptance. The difference between households with the least confidence and those being less up-to-date, undecided, or confident (rather or strongly agree) was statistically significant, with the outcome variable assuming full service provided online OR = 1.31 (95% CI 1.11 - 1.55), OR = 1.67 (95% CI 1.40 - 2.00), OR = 1.77 (95% CI 1.49 - 2.10), and OR = 2.94 (95% CI 2.21 - 3.91), respectively.

The use of health care services or admission to hospital of a member of a household in the preceding year did not influence acceptance of the use of the Internet for the provision of full health care services. The use of health care services in the preceding year was related to acceptance for at least partial delivery of health care services on the Internet.

## Discussion

The overall acceptance of the use of the Internet for the provision of full health care services has remained at the same level since 2007 when these items were first included in the questionnaire used for assessment of Polish households as part of the Social Diagnosis study (the percentage changed only from 28.1% in 2007 to 29.1% in 2011) [13]. In the subset of households included in the logistic regression model, the acceptance of the use of the Internet for full health care service provision was approximately 30% (2656/8915, 29.79%) and at least for access to information and downloading necessary forms, 45.09% (4020/8915). These levels are relatively high considering that only 61.32% of households surveyed had Internet access and the actual availability of eHealth services to patients in Poland is not extensive. On the other hand, the percentage of European citizens using the Internet for more interactive services than simply reading health-related information included in the survey of Kummervold et al [18] in 2007 was as high as 22.7%. The analysis of data from the 2007 Health Information National Trends Survey performed by Wen et al [19] showed that nearly 86% of adults in the United States rated electronic access to their personal health record as important, which is far higher than the degree of acceptance of at least partial delivery of Internet-based health care services among Polish households in 2011.

In our study, we did not analyze the actual use of eHealth services, but another survey indicates that the Internet was used to access health-related information by 23% of individuals in Poland in 2011 [20]. The percentage of the adult population accessing health-related information online in Poland appears to be at least 2 times lower than the percentage of households accepting to some extent the use of the Internet for health care service provision.

Relatively high acceptance of at least partial provision of Internet-based health care services is likely related to a general dissatisfaction with the health care system in Poland. Poland’s health care system has been undergoing a continuing process of reforms since the transition to a market economy in the early 1990s. The establishment of Regional Health Funds was one of the key changes in the late 20th century, followed by the return of a centralized funding of the health care system with the establishment of the National Health Fund in the early 21st century. Poland’s health care system is still based on public hospital services and outpatient care by private providers paid mainly from the Fund [21]. Access to health care services contracted by the Fund is subject to a queue system, and patients frequently wait months for certain specialist services. The gap between expectations for high quality care and access to services for patients and their families, and the actual capability of the system, remains a major source of frustration [22,23]. This creates an opportunity for providers or organizations developing new types of health services, especially originating from the eHealth domain.

Most surveys reported elsewhere about the acceptance or the use of eHealth services have been related to the experience of individuals representing a whole population or selected groups, such as patients with specific disorders. Nonetheless, for at least some of the predictors resulting from the multivariate logistic regression carried out for households in Poland, corresponding findings from other surveys may be indicated.

The disparities between rural and urban areas in the use of ICT have been described previously in Poland and in other countries [24-29]. In our analysis, the differences were significant only between households from rural and highly urbanized (> 200,000 inhabitants) areas. This result seems to confirm the general perception of a relatively poor information infrastructure in smaller cities and rural areas in Poland.

The lower acceptance of eHealth services was also revealed in households where the main income was from retirement or disability pensions. This observation illustrates the lower Internet penetration and literacy in the older strata of society, as well as lower access to modern communication technologies among people with disabilities. The relationship between the source of income and the acceptance for eHealth services is in line with general findings that professionally active people are more involved in using the Internet and computers than those who are retired or receiving disability pensions [30-34]. In addition to lower acceptance of the use of the Internet for health care services in households from rural areas, households with a main income from self-employment in agriculture showed lower acceptance in comparison to households where the main sources of income related to employment.

In our study, acceptance was consistently associated with higher household income per capita (comparison between lowest and higher quartiles). This finding is consistent with the results of studies performed in other countries [28,35,36]. In earlier surveys conducted in Poland, it was found that the presence of a child in the household is a driver of ICT use [24]. The results of logistic regression showed that this is also true in relation to acceptance of health care services provision through the Internet. The study performed by Hsu et al [35] in Northern California also revealed that households with children were more likely to have access to eHealth services provided by a service provider in this area.

The number of books in a household was included in the analysis in order to observe the influence the level of general literacy may have on the acceptance of the Internet as a tool for health care. Interestingly, the availability of at least some books in a household significantly affected the acceptance of using the Internet for health care services.

Our study also revealed that the factors related to ICT use in households were predictors of the acceptance of the Internet for the provision of health care services. The availability of a computer in a household, Internet access, and its duration in the household increased the level of acceptance. These findings seem to confirm the importance of the development of the information society on the acceptance of eHealth services. Similar results have been reported by other authors, both in relation to variables related to actual Internet use [27,36,37] and computer use [27]. The households included in the survey also expressed their opinion about familiarity and use of modern technologies. Higher confidence in this area was associated with higher acceptance of Internet use in the health care domain. This shows that acceptance of innovation and technology in itself results in a more open approach to new media for service provision in specific domains.

Interestingly, variables related to actual use of health care services by households had a limited impact on the acceptance of the use of the Internet for health care service provision. This was true for all 3 variables included in the logistic regression model apart from the use of health care services in the preceding year and increased acceptance of partial provision of health care on the Internet. The results of surveys performed in other countries indicate that households with at least 1 member with a high expected need for clinical services [35], include an individual with a history of cancer [26], actual use of health care services [27], current health problems in an individual or relatives [36,37], and have chronic disease or a poor perception of one’s own health [38] were associated with household or individual acceptance or use of the Internet for eHealth services. On the other hand, the survey carried out by Gracia et al [39] among older people in Spain revealed that Internet users had better self-rated health than non-users. A similar trend was described by Wang et al [29] who analyzed data from the 2001 National Household Travel Survey and found that individuals with medical conditions reported less frequent Internet use than those without medical conditions. In our study, satisfaction levels for the coverage of household health needs in comparison to 2 years ago had no impact on the acceptance of Internet-based health care.

The surveys which focused on individual opinions also showed that predictors of Internet acceptance or use for health-related activities included age [26,28,31,33], female gender [26,27,37,38], higher education level [28,31,33,38,40], marital status (being married or separated/divorced in comparison to being unmarried) [38], and helping another to deal with health issues [37]. These factors were not included in the multivariate logistic regression model used because it was based only on variables describing the households participating in the study.

### Limitations

The assessment of acceptance levels for eHealth services is usually undertaken in relation to individual respondents. In this study, the responses registered by the canvasser were given on behalf of the whole household. Thus, the selection of potential factors which could influence the household’s acceptance of using the Internet as a tool for delivering health care services was made from the variable which could characterize household’s readiness to accept eHealth services.

The use of the concept of household acceptance in relation to eHealth services may be misleading because it is likely that not all members of a household share a common view and opinions may be diverse. On the other hand, the use of health care services usually depends on the decision of the individuals responsible for the household and their perception probably dominated in the views expressed during the canvasser interviews.

The main objective of the Social Diagnosis study was not focused on the eHealth field. Instead, it was oriented toward general issues about the household economic status and individual’s quality of life. Furthermore, the aspects of the use of ICT and the phenomenon of social divide were targeted. The strategy employed in our paper was to assess the acceptance of eHealth in Polish society using the data available from a study encompassing the whole population in a well-established and methodologically proven study.

The number of cases included in the multivariate logistic regression model presented in our paper was reduced from 12,386 to 12,015 due to missing values of key variables used to define outcome variables in the model. Furthermore, households that did not anticipate using health care services in the near future were excluded, and cases with missing values for predictors used in the model were omitted. As a result, data from 8915 households were used in the logistic regression model.

The number of missing values for outcome variables was not high (3.00%, 371/12,386), and its significance is difficult to assess due to a lack of information about potential reasons for the lack of response. The exclusion of households that did not anticipate using health care services in the near future (13.73%, 1700/12,386) was a potential source of bias in the results of the analysis. It is possible that households without sufficient levels of understanding of using the Internet for health care services provision, or those that do not accept such use, may have selected this response in order to hide their actual position. Thus, the exclusion of this group of households could suggest that a higher number of households actually accept using the Internet for this purpose. As for cases excluded from the final logistic regression model due to missing values in predictor variables (11.30%, 1400/12,386), the highest drop-out rates were related to the lack of data about monthly household net income per capita (4.28%, 530/12,386), the perception of current economic status of a household (3.17%; 393/12,386), the opinion on satisfying health care needs of a household (2.28%; 282/12,386), and hospitalizations of household members (0.99%, 123/12,386).

Interpreting missing values within these variables is difficult because of the association with the acceptance of Internet use. It is possible that households with extremely low or high monthly income rates per capita could be more prone to withholding information about their actual income. This could also be valid for the relatively high number of missing values in the variable related to the opinion of a household about its economic status in comparison to the preceding period. Assuming that the number of households with very low incomes in Poland is significantly higher than the number of households with high incomes, and because poverty is linked to a lower acceptance of ICT technologies, the relationship between income and the acceptance of the Internet for health care services is likely to be closer than shown.

As for the variables related to the opinion about satisfying health care needs of the household and hospitalization of a household member in the past year, any potential bias in the assessment of final results is not clear. These variables did not have a significant effect on the acceptance of using the Internet for health care services provision. The households that were reluctant to respond to these items could be generally dissatisfied with health care services and did not think that the Internet could provide a working solution. It is also possible that a lack of response to this item was due to the household not having used health care services extensively in the preceding period. Furthermore, hospitalization of a household member in the preceding months is likely to have resulted in focusing on the current situation instead of emerging solutions. However, the net effect of missing values within these variables is not clear.

### Conclusions

The acceptance of health care services via the Internet was higher in households from larger cities, with stable income from an employee salary, as well as with higher income levels per capita. Furthermore, general computer and Internet use in the household influenced the perception of eHealth. Paradoxically, the use of health care services or the level of satisfaction with the coverage of the household’s health needs exerted a limited influence on acceptance of Internet-based health care.

## Abbreviations

EU: European Union
ICT: information and communication technology
OR: odds ratio
